# CLSoilMaps: A national soil gridded database of physical and hydraulic soil properties for Chile

**DOI:** 10.1038/s41597-023-02536-x

**Published:** 2023-09-16

**Authors:** Diego I. Dinamarca, Mauricio Galleguillos, Oscar Seguel, Carlos Faúndez Urbina

**Affiliations:** 1 Facultad de Ingeniería y Ciencias, Universidad Adolfo Ibáñez, Diagonal las Torres 2640, 7941169 Peñalolen, Chile; 2 Center for Climate Resilience Research (CR2), Blanco Encalada 2002, 8370449 Santiago, Chile; 3 Data Observatory Foundation, ANID Technology Center No. DO210001, Eliodoro Yáñez 2990, 7510277 Providencia, Chile; 4 Facultad de Ciencias Agronómicas, Universidad de Chile, Santa Rosa 11315, 8820000 La Pintana, Chile; 5 Escuela de Agronomía, Facultad de Ciencias Agronómicas y de los Alimentos, Pontificia Universidad Católica de Valparaíso, San Francisco S/N, 2260000 Quillota, Chile

**Keywords:** Geomorphology, Hydrology

## Abstract

Spatially explicit soil information is crucial for comprehending and managing many of Earth´s processes related to carbon, water, and other biogeochemical cycles. We introduced a gridded database of soil physical properties and hydraulic parameters at 100 meters spatial resolution. It covers the continental area of Chile and binational basins shared with Argentina for six standardized depths following the specifications of the GlobalSoilMap project. We generated soil maps based on digital soil mapping techniques based on more than 4000 observations, including unpublished data from remote areas. These maps were used as input for the pedotransfer function Rosetta V3 to obtain predictions of soil hydraulic properties, such as field capacity, permanent wilting point, total available water capacity, and other parameters of the water retention curve. The trained models outperformed several other DSM studies applied at the national and regional scale for soil physical properties (nRMSE ranging from 6.93% to 15.7%) and delivered acceptable predictions (nRMSE ranging from 10.4% to 15.6%) for soil hydraulic properties, making them suitable for countless environmental studies.

## Background & Summary

Soil is a vital element of the functioning of the biosphere, being the structural base for the exchanges of matter and energy between the atmosphere, lithosphere, and hydrosphere^1^. Soils sustain life by being at the center of carbon, water, and other biogeochemical cycles. Therefore, soils are recognized for providing different services to humanity, which need to be guaranteed to achieve sustainable development. The international community has recognized the importance of soils by including them in at least twelve of the seventeen United Nations Sustainable Development Goals^2^.

Soil’s capacity to provide ecosystem services is tightly linked to its physical and chemical properties, biodiversity, and the interactions between these components, which can be modified mainly by anthropic use and management practices, especially in critical development areas like the agricultural and forestry sectors^3^. Of great relevance are those services related to water provision and regulation where soil physical properties play a fundamental role, given that they represent the interface where the atmosphere meets the Earth and modulates fluxes of matter and energy within the continuum that ranges from substrate underlying soils to the top of the atmosphere^4^. Indeed, soil physical properties and their soil hydraulic parameters may be relevant in ecosystem and hydrological modeling since many processes implemented in such tools directly depend on those properties^5,6^. Since soil texture and bulk density determine the capacity of soil to store and infiltrate water, both properties are linked to runoff control^7,8^, CO_2_ efflux^9^, and organic matter mineralization^10–12^, among other natural processes. In many situations, this information is extrapolated from a global database or obscured by calibration procedures introducing more considerable uncertainties into simulations of carbon and water fluxes variables. These issues justify the need for high-quality soil information to support management strategies^13^, especially those related to water management.

A few years ago, soil spatial information was limited to soil class maps, where different types of soils are depicted as polygons^14^, created using conventional mapping methods. These models assumed that soils within the same class had low variability and that the changes between classes were discrete, separated by polygon borders. Consequently, this type of map failed to reveal details about intrapolygonal variation within each class, leading to a lack of precision regarding soil attributes^15^.

Given the limitations of conventional mapping methods, another geospatial technique has been developed to estimate the continuous variations of soils in space, at finer scales, which can more adequately represent soils processes known as digital soil mapping (DSM). Most DSM approaches apply the SCORPAN model^16^, formalized in the equation *S* = *f(scorpan)*, where *S* is a soil class or attribute, each letter of the *SCORPAN* acronym represents a soil-forming factor (s: soil, c: climate, o: organisms, r: relief, p: parent material, a: age/time, n: space/spatial position) and *f()* is a quantitative empiric function that relates soil properties to their soil forming factors.

There is a rich offer in geospatial data that can nourish prediction models and from which thousands of soil-forming factors-related covariates can be derived. Among the most utilized predictors are maps of existing soil properties, mean annual temperature and precipitation, remote sensing images, elevation, land attributes, and geological maps^17^. The covariates used in the predictive models vary among studies, and there is no consensus about the optimal quantity. However, it is assumed that more covariates imply more precise results, even when the method selects only a subset to obtain the predictions^18^.

Naturally, the availability of soil observations is an important limiting factor to applying DSM, given that a soil survey and subsequent laboratory analysis is a costly and time-consuming task^19^. This issue, added to the fact that some soil attributes are more challenging to measure than others, results in soil databases that often lack valuable information like water retention properties. They are usually obtained from pedotransfer functions (PTF) to predict soil attributes such as field capacity, permanent wilting point, or total available water capacity from other soil attributes that are more easily measured or that demand fewer resources to obtain like soil particle size fractions or bulk density.

Soil physical properties and hydropedological properties gridded products are now available for the entire world, usually at 250 m (“https://soilgrids.com”, last access Oct-28; “https://openlandmap.org”, last access Oct-28); however, only a limited set of local observations were considered for Chile (e.g., 26 soil profiles for bulk density and 69 for textures in the WoSIS database used for SoilGrids^20^), thus introducing possible uncertainties. In Chile, the widely used soil spatial information pertains to soil class maps prepared on a 1:20000 and 1:50000 scale, developed by the Center of Natural Resources Information (CIREN) by conventional soil mapping techniques based on more than six hundred soil samples located mainly in agricultural and grassland systems^21^. These data and other compiled soil information were used to develop gridded soil maps for different physicochemical properties (organic carbon, field capacity, permanent wilting point, bulk density, clay and sand particle size fraction, and pH) with a resolution of 100 m at the national level as an input to the GlobalSoilMap project^21,22^ although with moderate precision and not publicly available. Despite its inherent value as a pioneer study devoted to Chile only, more significant uncertainties are expected since it was based on data that were mainly sampled in agricultural lands primarily located in flat areas under gentle climate conditions, all of which is far from the reality of the country’s natural variability characterized by mountainous topography and harsh weather ranging from extreme desert, rainy and polar climates^23^. Therefore, such soil-derived products can have significant consequences on hydrological applications and forestry studies, including natural and planted ecosystems that require a precise characterization capturing all kinds of topo-climatic conditions.

The present research provides a new gridded product of soil physical and hydraulic properties which accounts for the natural variability of the country, using a newly compiled soil database for the national territory that includes soil profiles from mountainous ecosystems and hostile weather in addition to agricultural and forestry samples. DSM techniques coupled with more than two hundred potential environmental covariates are used to model clay, sand content and bulk density. Also, pedotransfer functions are used to obtain maps of field capacity, permanent wilting point, available water capacity soil saturated hydraulic conductivity, among other soil attributes, from the soil physical properties modeled with DSM. All maps were generated for six standard depths following the GlobalSoilMap project protocols at 0.001 degrees of spatial resolution (near 100 m).

## Methods

### Soil data

**Fig. 1:**
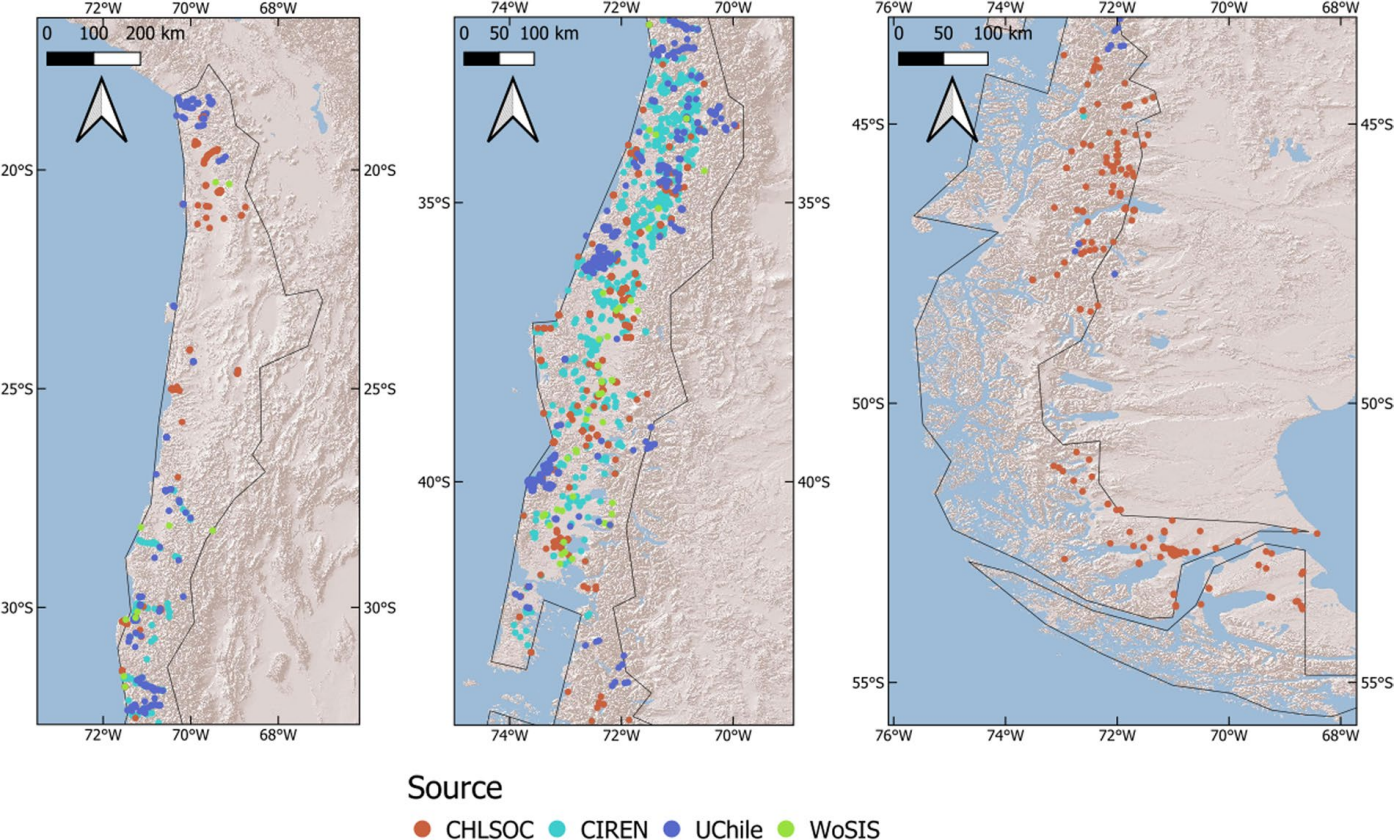
Spatial distribution of soil profiles used in the mapping procedure.

The soil profile database used corresponds to a compilation of diverse published sources^20,24–34^, including a newly compiled database called ChSPD^35^. The data was classified into four categories, shown in Fig. 1. Data from CIREN consist of 46% of texture data and 21% of bulk density and are distributed mainly in soils with agricultural aptitude. On the other hand, data from the Laboratory of soil physics at the University of Chile (UChile) represents 41% for texture, 30% for bulk density and 90% of the hydraulic soil atributes. The Chilean Soil Organic Carbon Database (CHLSOC) represents 8%, 48% and 10% for texture, bulk density and soil hydraulic properties data (Table 1). These two databases are compilations of different authors and provide data from soils in diverse natural ecosystems such as the Atacama Desert, Andes Mountains, and Patagonia, in addition to native forests and forestry plantations in the south-central zone of the country. Only a few samples have the sampling year, so this variable was not considered for modeling, which is assumed not to be an inconvenience given the invariant nature of the soil properties chosen.

Soil samples were standardized at six depths 0–5, 5–15, 15–30, 30–60, 60–100, and 100–200 cm, according to GlobalSoilMap project specifications^22^, using an equal-area splines method^36^. Given the resolution of 0.001 degrees used to generate the predictive maps, some pixels contained more than one soil profile sample. To deal with the redundant information that would be generated in the regression matrix, all the soil profiles that were inside the same pixel were averaged after the standardization. The resulting dataset was then overlayed with the environmental covariates to generate the regression matrix.

### Environmental covariates

We selected predictors according to the soil formation factors proposed in the SCORPAN model. Table 2 shows a summary of all the predictors considered. We resampled all environmental covariates with the final resolution of the soil maps (0.001 degrees) before overlaying soil profiles and training the random forest models. The methodology to obtain and process the predictors is detailed in the following paragraphs.

#### Climate

Climate predictors were obtained from gridded precipitation and maximum, minimum, and mean temperatures prepared for continental Chile by the Center for Climate and Resilience Research (CR2). These products have a spatial resolution of 0.05 degrees (near 5 km) and have daily and monthly images from 1979 to 2020^37^. Only the period from 1979 to 2009 was considered to obtain metrics related to the typical historical climate behavior, without considering the last ten years of megadrought^38^. Several environmental predictors were calculated, such as monthly long-term precipitation, mean, maximum, and minimum temperature, aside from 21 bioclimatic indexes that give information about the annual behavior of climate and average conditions and its seasonal intra-annual variation^39^. All these covariates were resampled to 0.001 degrees using a bilinear method.

#### Soil and organisms

Surface reflectance measured by Landsat 8 was considered a proxy for obtaining soil and organism predictors. To create the Landsat 8 composite for the whole study area, an application developed for Google Earth Engine (GEE)^40^ was used. This app considers four criteria for determining the best-quality pixel among images. These criteria are explained briefly next: (1) Year of image, where the highest weight was assigned to the scenes in the middle of the period (1979–2009), and the weight decreases linearly to the limits of the year interval; (2) The period of the composite (e.g., summer months), greater weight was assigned for the images at the half of the period, decreasing in a bell shape towards the limits of the period; (3) Distance to clouds or cloud shadows, where the highest weight was assigned to the images that are farther than 1500 m from a cloud or cloud shadow, decreasing with distance according to a sigmoid function; and (4) The median reflectance value of the pixel, which was applied to reduce oversaturation or interference of clouds and cloud shadow. Here, the weight was assigned according to the near-infrared (NIR) band values, where pixels from the median of the distribution have the highest weight, decreasing linearly toward the limits of the distribution. For every scene pixel, the weighted weight was calculated, and the final composite was created with the pixels with the highest weight.

The study area represented as an image composite was divided into two zones due to certain environmental limitations. In January and February 2017, the country’s central zone was affected by mega-wildfires, which modified the ordinary conditions of the land^41^. For this reason, from 41.44 to 17°S, summer images were considered between December 21 and March 21 from 2014 to 2016. South of 41.44°S, the main limitation was the presence of clouds, so the period of the year had to be increased from 2014 to 2021, considering the same summer period. The final composite was downloaded from GEE and resampled to 0.001 degrees using the nearest neighbor method.

Missing pixels on the final composite were filled using a focal filter with a 5 × 5 window. Several spectral indexes were calculated from the composite: the Green Normalized Difference Vegetation Index (GNDV)^42^, Green-Red Vegetation Index (GRVI)^43^, Grain Size Index (GSI)^44^, Normalized Difference Vegetation Index (NDVI)^45^, Soil Adjusted Vegetation Index (SAVI)^46^; images of brightness, greenness, and wetness were calculated using a Tasseled Cap transformation^40^; and soil enhancement ratios: red/green, red/swir2, swir1/swir2 that augment the presence of some exposed soil minerals^47^. Finally, textural metrics of contrast, variance, entropy, dissimilarity, and homogeneity (inverse difference moment) were calculated using the grey-level co-occurrence matrix (GLCM) for all the previous spectral indexes^48^.

#### Relief

The predictors in this category were obtained from the Shuttle Radar Topography Mission (SRTM) digital elevation model of 30 m resolution, which was downloaded from the GEE platform^49^ and resampled to 0.001 degrees using the nearest neighbor method. Several first and second-degree topographic derivatives were calculated using open-access software SAGA GIS^50^ which can be seen in Table 2.

#### Depth

Depth was added as a predictor following the methodology proposed by Akpa *et al*.^51^ where the mean of each horizon was added as a covariate (e.g. 2.5 for 0–5 cm horizon, 10 for 5–15 cm horizon, and so on). This allowed us to create 3D models that allowed us to train one single model that could predict on all soil horizons at once.

### Covariate selection

Given the large number of covariates generated, it was necessary to make a preselection to obtain more parsimonious models. For this, we used an automated method of variable selection based on Random Forest implemented in the R package VSURF^52^, which takes advantage of this model’s capacity to estimate the covariates’ predictive importance (PI). The VSURF capabilities have proven effective in remote sensing-based predictive modeling in other contexts such as biomass^53^ and post-fire litter^54^ mapping.

**Fig. 2:**
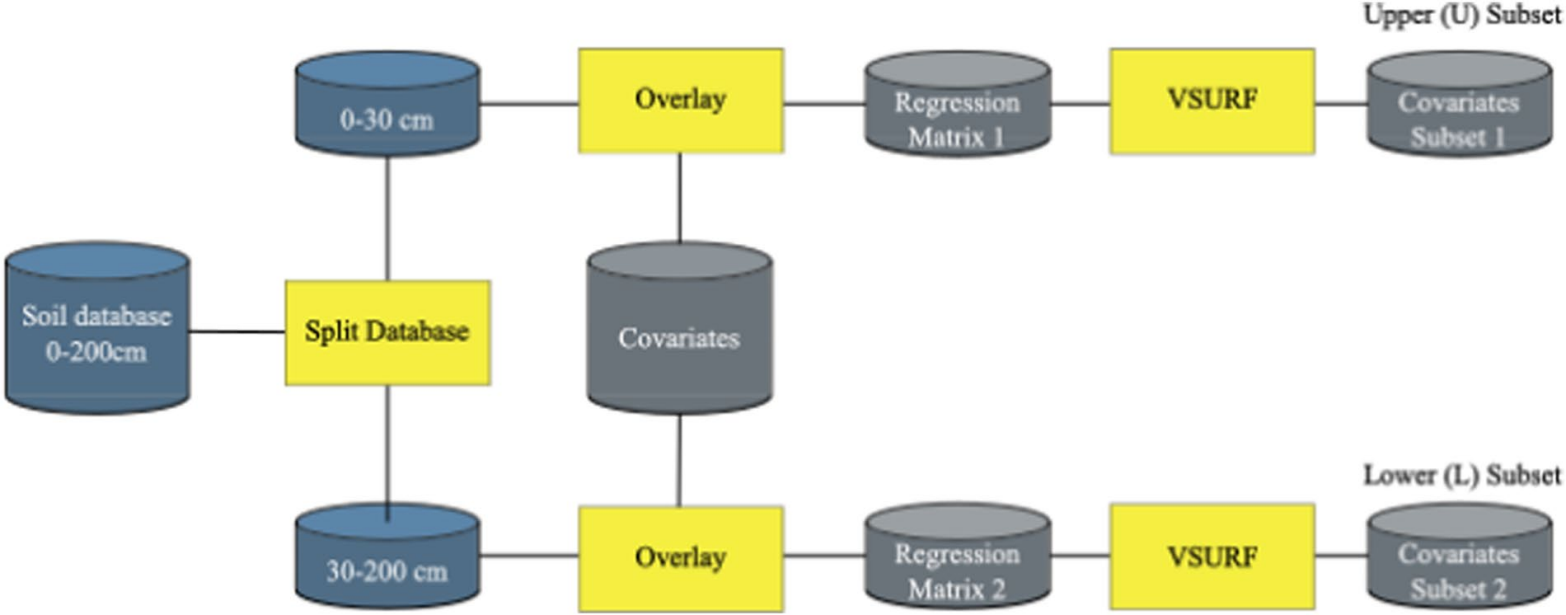
Variable selection workflow. Two covariate subsets are obtained for each soil attribute, one for each of the soil database splits.

First, we split the database in two: one half with soil data from 0–30 cm (horizons 0–5, 5–15, and 15–30 cm) and the other half with data from 30–200 cm (horizons 30–60, 60–100, 100–200 cm). Then each split was overlayed with the complete set of covariates already resampled to 0.001 degrees to create two regression matrices used as inputs for the VSURF function to select the most important predictors. The result of this selection process was two subsets of covariates: upper (U) and lower (L) for each soil physical property (Fig. 2).

Once both subsets were obtained, a correlation filter was applied to all the continuous variables so that each pair of predictors with a correlation coefficient larger than 0.6. The variable with the lowest variable of importance, estimated by the VSURF function, was removed from the set. Then, depth was forced as a covariate in all models to ensure that the models could predict at any standardized depths.

#### Random forest model tuning

To link observed soil values with the environmental covariates, Random Forest (RF) was used as the predictive model^55^, given its processing speed, resistance to overfitting, and ability to handle high dimensionality and categorical and quantitative predictors. This model has some parameters that can be adjusted to get a better fit. These parameters were the number of trees (ntree), the number of predictors selected at each split (mtry), and the minimum number of values for each branch or terminal node (nmin). These three parameters were adjusted for each modeled variable using the tune grid function of R package *tune*^56^, choosing the values that resulted in the lowest RMSE.

### Model training and validation

Training Random Forest models started by splitting the database into 75% for the training set and 25% for external model validation. Then the training set was sampled randomly with repetition in a bootstrap process with one hundred iterations. In each iteration the number of values sampled was 75% of the total training set. With the sampled data, a random forest model was trained using as covariates the Upper and Lower subsets obtained on the covariates selection process, resulting in two different trained models per iteration. Repeating this process one hundred times, we got 100 models per soil attribute and covariates set. The 200 models were then validated against the 25% of the external validation set which allowed us to measure the generalization of the models using data that was never included in the training process. The validation metrics calculated were RMSE (Eq. 1), PBIAS (Eq. 2), R^2^ (Eq. 3)., and nRMSE (Eq. 4), differentiating the results for each soil standardized horizon. The model with the highest R^2^ was selected for each horizon to generate the final soil maps.

1 $$RMSE=\sqrt{\left(\frac{{\sum }_{i=1}^{n}{({y}_{i}-\bar{y})}^{2}}{n}\right)}$$

2 $$PBIAS=100\ast \frac{{\sum }_{i=1}^{n}({\widehat{y}}_{i}-{y}_{i})}{{\sum }_{i=1}^{n}({y}_{i})}$$

3 $${R}^{2}=\frac{{\sum }_{i=1}^{n}({\widehat{y}}_{i}-\bar{y})}{{\sum }_{i=1}^{n}({y}_{i}-\bar{y})}$$

4 $$nRMSE=\frac{RMSE}{\left({y}_{max}-{y}_{min}\right)}$$

To evaluate model uncertainty, we calculated the prediction interval coverage probability (PICP) suggested by^57^. This value corresponds to the percentage of samples contained inside the boundaries of a prediction interval, given a level of confidence. The procedure consisted of estimating the prediction interval of each response using different confidence levels for each model. Then for each model, precision maps were generated^58^ showing the proportion of observed data within the prediction interval at different confidence levels.

### Generating digital soil maps

As a result of the bootstrap process, we obtained 200 trained models, two for each iteration. With each of those models one map was generated for each property and standardized depth, getting a distribution of 100 values for every pixel. The final map for each property and depth was generated by calculating the mean of the distribution. Also masks of urban areas, water bodies (available at “https://www.bcn.cl/siit/mapas_vectoriales”) and glaciers (available at “https://dga.mop.gob.cl/estudiospublicaciones/mapoteca/Paginas/Mapoteca-Digital.aspx”) were applied to the final maps since these types of cover are not considered soil.

Then with the mean of sand and clay, silt content was calculated using Eq. 5:

5 $${\rm{Silt}} \% =100-\left({\rm{Sand}} \% +{\rm{Clay}} \% \right)$$

Since sand and clay content were estimated independently, the sum of both gave values above 100%, so specific pixels yielded a negative silt content. All the negative pixels were replaced with a focal filter by the mean of the neighboring pixels using a 5 × 5 window.

Next, to evaluate the uncertainty of the models, we calculated prediction intervals with a 90% confidence level, estimating the upper and lower limits using Eq. 5^21^.

6 $${\rm{PI}}=\bar{{\rm{x}}}\pm 1.645\sqrt{{{\rm{\sigma }}}^{2}+{\rm{MSE}}}$$

Where $\bar{x}$ and ${\sigma }^{2}$ correspond to the mean and variance of the Bootstrap iterations, and MSE is the quadratic mean error associated with the 100 trained models. The limits of the prediction intervals were capped to the actual boundaries for the selected physical properties (e.g., clay and sand content between 0 and 100%, and a bulk density greater than 0) to prevent intervals with unreal limits.

### Map of soil textural classes

We standardized the maps of clay, silt, and sand (considering that the three particles add up to 100) to obtain the textural classes map, for example to standardized sand content:

7 $$San{d}_{c}=\frac{Sand}{\left(Clay+Silt+Sand\right)}\cdot 100$$

where Sand_c_ was the corrected sand content. Once all three particles were standardized, we used the R package soil texture (Moeys, 2018) to get a textural classification map according to the USDA classification system.

### Generating maps with PTFs

In addition to the soil properties modeled, soil water retention, and available water capacity were obtained using Rosetta V3^59^. Rosetta is a PTF that estimates water retention parameters in van Genuchten’s (1980) equation:

8 $$\theta (h)=\left\{\begin{array}{cc}{\theta }_{r}+\frac{{\theta }_{s}-{\theta }_{r}}{{[1+{| \alpha h| }^{n}]}^{m}}, & h\le 0\\ {\theta }_{s}, & h > 0\end{array}\right.$$

We obtained the soil water content at field capacity (FC; h = 330kPa), and the permanent wilting point (PWP; h = 15000kPa) using the parameters estimated by Rosetta V3. Next, the available water capacity (AWC) was calculated using the following equation:

9 $$AW{C}_{h}=(FC-PWP)\ast {H}_{thickness}$$

Where *AWC*_*h*_ corresponds to the available water capacity for each soil horizon, FC and PWP are the field capacity and permanent wilting point estimated by Rosetta, and H_thickness_ is the thickness of the standardized horizon in mm (namely 50, 100, 150, 300, 400, 1000 mm for the six standardized horizons 0–5, 5–15, 15–30, 30–60, 60–100, 100–200 cm). The total available water capacity for the whole soil profile was given by the sum of AWC values across all soil horizons.

Maps of FC and PWP were validated against measured values (Table 1) that were standardized to the six soil horizons using equal area splines with the same methodology as the physical properties modeled with DSM.

**Table 1:** Number of soil profiles and soil samples per source and soil attribute. BD = Bulk Density, PWP = Permanent Wilting Point, FC = Field Capacity.

Soil attribute	Source	N of profiles	N of samples	% of samples
Clay, Silt and Sand	CHLSOC	98	267	8%
	CIREN	557	2269	46%
	UChile	496	1612	41%
	WoSIS	61	318	5%
	Total	1212	4466	69%
BD	CHLSOC	851	1427	48%
	CIREN	365	1476	21%
	UChile	531	1701	30%
	WoSIS	18	112	1%
	Total	1765	4716	100%
PWP	CHLSOC	42	194	10%
	CIREN	0	0	0%
	UChile	397	1354	90%
	WoSIS	1	9	0%
	Total	440	1557	100%
FC	CHLSOC	42	194	10%
	CIREN	0	0	0%
	UChile	399	1368	90%
	WoSIS	1	9	0%
	Total	442	1571	100%

**Table 2:** SCORPAN predictors.

Product	Resolution	Type	Predictors
CR2MET Precipitation and Temperature (mean, max and min) grids	5 km	Climate	Isothermality, Max temperature of warmest month, Mean Precipitation of coldest quarter, Mean precipitation of driest quarter, Mean precipitation of warmest quarter, Mean Precipitation of wettest quarter, Mean temperature of coldest quarter, Mean temperature of driest quarter, Mean temperature of warmest quarter, Mean temperature of wettest quarter, Min temperature of coldest month, Precipitation of direst month, Annual mean precipitation, Annual standard deviation of precipitation, Precipitation seasonality, Total annual precipitation, Precipitation of wettest month, Temperature annual range, Temperature mean diurnal range, Temperature seasonality Coefficient of variation, Temperature seasonality Standard deviation, Long term mean monthly precipitation, Long term mean monthly temperature, Long term maximum monthly temperature, Long term minimum monthly temperature.
Landsat 8 Surface Reflectance	30 m	Soil and Organisms	GNDVI, GRVI, NDVI, SAVI, GSI, Brightness, Greenness, Wetness, red/green, red/swir1, swir1/swir2
SRTM	30 m	Relief	Elevation, Catchment area, Catchment slope, Convergence Index, Cross sectional curvature, Plan curvature, Tangential curvature, Diurnal anisotropic heat, Flow direction, Flow length, Mid slope position, MRRTF, MRVBF, Normalized Height, Saga Wetness Index, Slope Height, Standardized Height, Stream power index, Terrain surface classification*, Terrain surface texture, Topographic position Index (1000 m and 5000 m), Terrain ruggedness index, Valley depth

*Categorical variable.

### Spatial analysis

We analyzed the spatial variability of the CLSoilMaps products of textures, bulk density, and textural classes according to a map of the ecoregions^60^. The ecoregions of the Sechura Desert and Central Andean Puna were added to the Atacama Desert and Central Andean Dry Puna because they span less than 1% of the total national territory, and there needed to be more soil samples within these areas. We compared the similarity of the frequency distribution of the observed values against CLSoilMaps and SoilGrid^61^ products for each ecoregion.

## Data Records

CLSoilMaps can be downloaded from the Zenodo repository at 10.5281/zenodo.7464210^62^. This repository has different zip files that contains different groups of soil maps:

1. SoilMaps_Mean: Maps of soil physical properties mean across 100 bootstrap iterations. Bulk density, clay, sand. Silt maps were generated using Clay and Sand values (see Methods for details).
2. PIRange: Maps of soil physical properties uncertainty estimated with 100 bootstrap iterations. These are only available for bulk density, clay and sand.
3. ROSETTA_MEAN: Mean maps of soil hydraulic properties generated with Rosetta V3. There are also available maps for each parameter of the Van Genuchten equation.
4. ROSETTA_SD: Standard deviation of soil hydraulic properties generated with Rosetta V3
5. Textural_Classes: Maps of soil textural classes using USDA classification scheme.

Each file contains the information for one of six standardized horizons which are: 0–5, 5–15, 15–30, 30–60, 60–100, and 100–200 cm. All files specify to which standard horizon it corresponds in its filename. Details for every soil attribute can be found on Table 3.

**Table 3:** Summary of soil attributes available in CLSoilMaps.

Soil attribute	File abbreviation	Description	Units
Bulk density	Bulkd	Bulk density of the fine fraction	g/cm^3^
Clay	Clay	Clay content	%
Sand	Sand	Sand content	%
Silt	Silt	Silt content	%
Field Capacity	FC	Field capacity at 330kPa	cm^3^/cm^3^
Permanent Wilting Point	PWP	Permanent wilting point at 15000kPa	cm^3^/cm^3^
Available Water Capacity	AWC	Available water capacity as 100*(FC-PWP)	mm
Total Available Water Capacity	Total_AWC	Sum of AWC across all depths	mm
Available Moisture	AvMoist	Available Moisture as FC-PWP	cm^3^/cm^3^
*θ*_*r*_	theta_r	residual water content	cm^3^/cm^3^
*θ*_*s*_	theta_s	saturated water content	cm^3^/cm^3^
*α*	alpha	“alpha” shape parameter	1/cm
npar	n	“n” shape parameter	—
Soil Hydraulic Conductivity	ksat	saturated hydraulic conductivity	cm/day

## Technical Validation

### Covariate selection

**Fig. 3:**
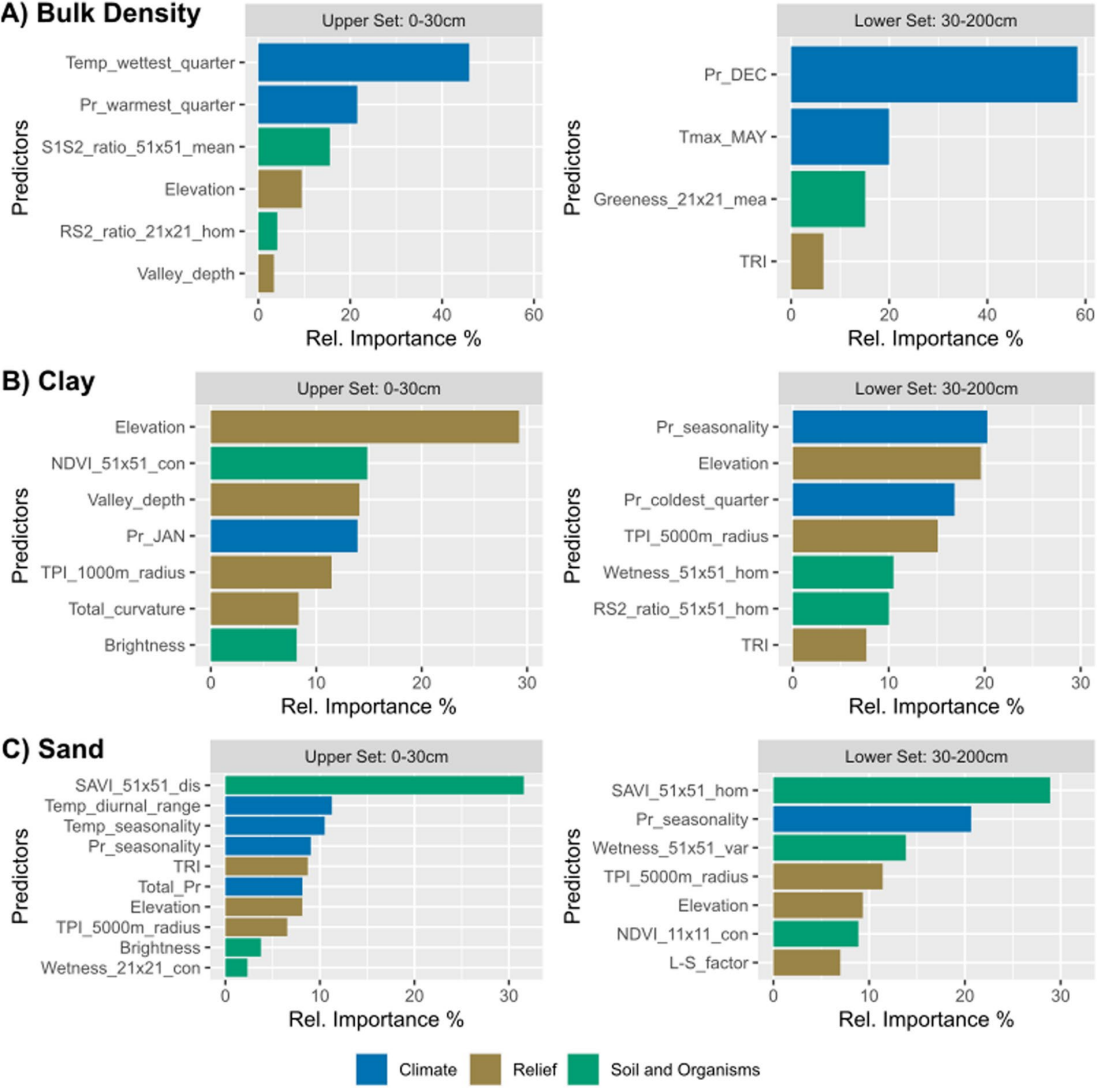
Covariates selected as predictors for each soil attribute. The predictors selected by the VSURF function were filtered, eliminating all the predictors with a correlation greater than 0.6. The upper and lower sets correspond to the portion of the soil database used for selecting the covariates. Pr: Precipitation, Temp: Temperature, SD: standard deviation, CV: Coefficient of variation, TPI: Topographic Position Index.

The process of variable selection included a total of 269 predictors. For each property, two sets of covariates were obtained (Fig. 3) from each database splits described in the methodology. The initial pool of predictors was reduced with the VSURF and correlation filter to only 6–8 variables per set.

Climate variables were the most important type of predictors for bulk density. Temperature seasonality was the most important predictor in the U set, and monthly precipitation of December for the L set, with more than 40% relative importance. In comparison, topographic, soil, and organism predictors had much less predictive power for both the U and L subsets. In contrast, topographic variables showed greater predictive importance for clay, especially elevation, with relative importance close to 30% for the U set. Climate variables for clay were all related to precipitation: mean precipitation of January, precipitation seasonality, and precipitation in the coldest quarter, and show greater predictive importance on the L set. Moreover, the top predictors for sand were related to soil, organisms, and climate variables. The SAVI vegetation index was the most important predictor in both sets, with relative importance close to 30%, while climate variables such as temperature diurnal range and temperature and precipitation seasonality follow on predictive importance.

For all the sets, most spectral variables selected correspond to textural metrics of the vegetation indexes. These textural metrics add contextual information from the neighbourhood of the variable; in this sense, it seems that at the scale of Chile, textural metrics generated with wider windows provide more useful information than smaller ones, given that 7 out of 13 of the selected predictors were calculated with a 51 × 51 neighbourhood window.

### Model accuracy and uncertainty

**Fig. 4:**
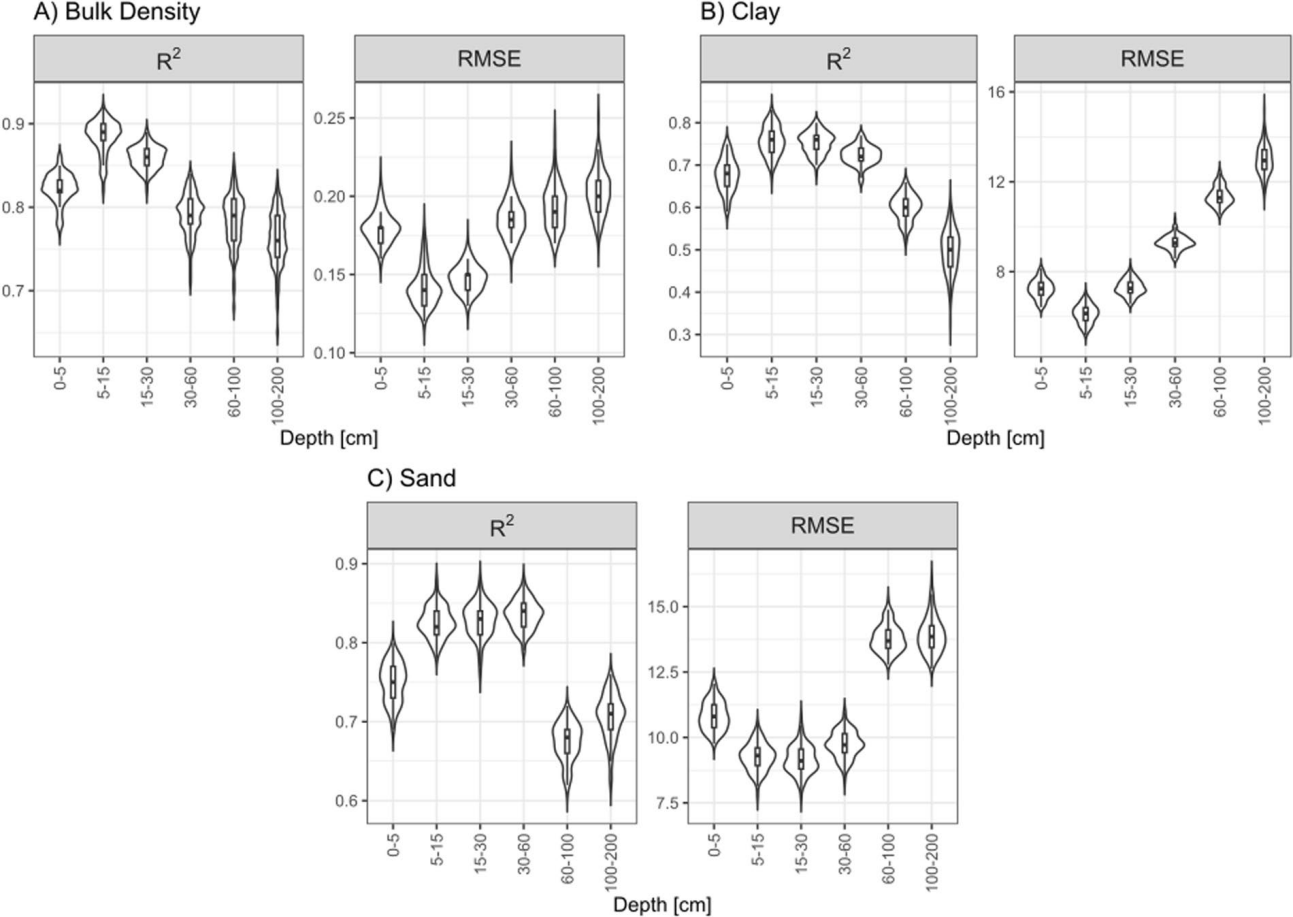
Performance metrics for the final selected models. Beanplots show the distribution of accuracy metrics (RMSE) and precision (R2) for 100 bootstrap simulations for each property and depth.

**Fig. 5:**
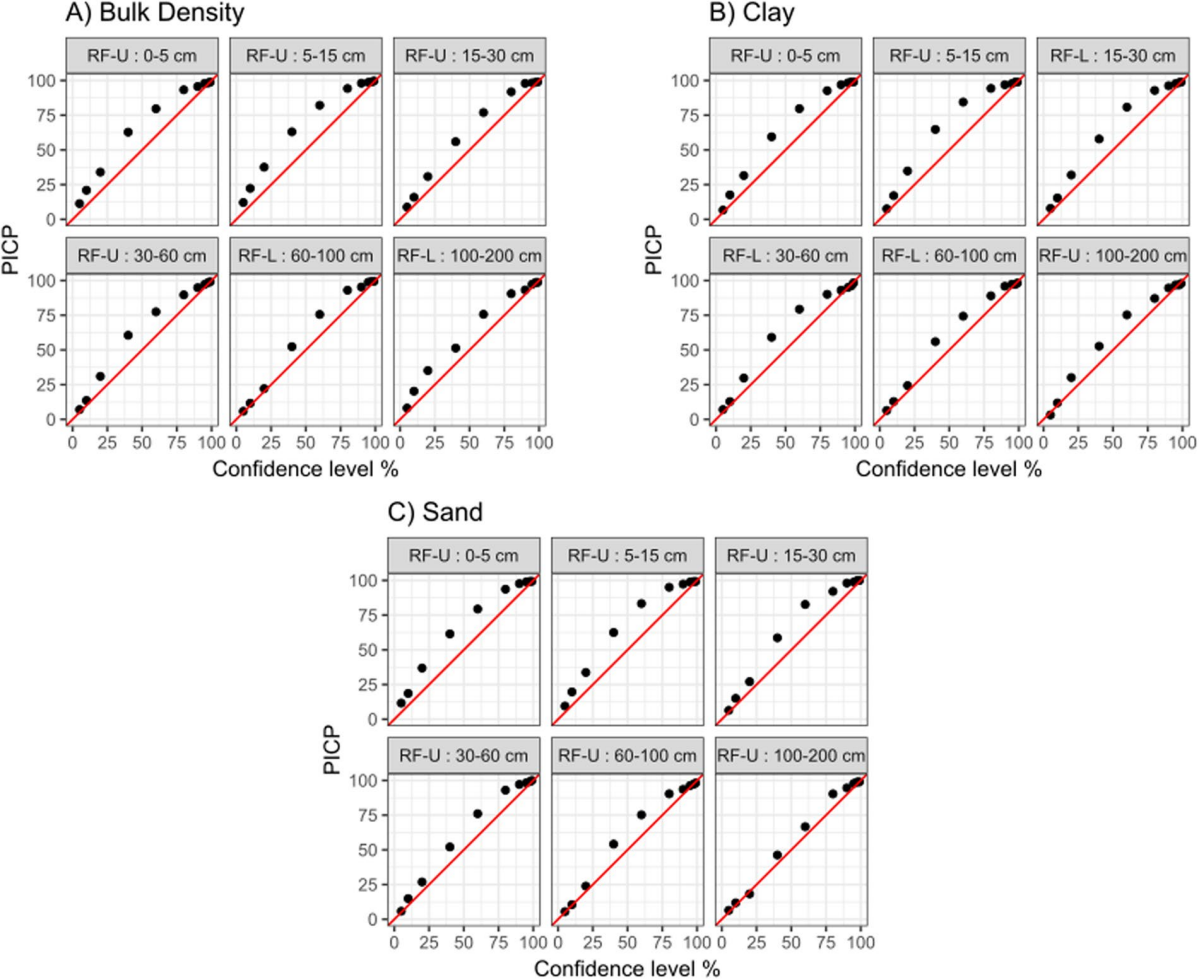
Prediction interval coverage probability (PICP) for the final selected models.

R2 values ranged from 0.76 to 0.88 for bulk density, 0.50 to 0.76 for clay, and 0.67 to 0.84 for sand. RMSE values ranged from 0.14 to 0.20 kg/m3 for bulk density, 6.11 to 12.99% for clay, and 9.16 to 13.91% for sand. nRMSE showed that bulk density was the most precise soil property modeled compared to sand and clay. Bias was not significant for bulk density and sand, with PBIAS values ranging from −0.90 to 0.20% and −3.82 to 2.47%, respectively, and for clay, it presented a minimum of −7.17% at 100–200 cm. However, it showed a similar PBIAS to sand at other depths (−5.93 to 3.90%). The distribution of R2 and RMSE values across the 100 bootstrap iterations showed a decrease in the performance with depth and a higher range in the distribution of the performance metrics (Fig. 4, details in Table 4). Regarding uncertainty, for a 90% confidence interval, PICP values were between 0.93–0.98 for bulk density, 0.94–0.98 for sand, and 0.93–0.97 for clay (Fig. 5, details in supplement 1). This deviation above the 1:1 line indicates that observed values fall into the prediction intervals more times than they theoretically should, which means that prediction intervals are too broad.

**Table 4:** Mean validation metrics across 100 bootstrap iterations.

Soil Attribute	Model	Depth	RMSE	nRMSE	R^2^	PBIAS
Bulk Density	RF-U	0–5	0.18	8.96	0.82	0.84
	RF-U	5–15	0.14	6.93	0.88	−0.04
	RF-U	15–30	0.15	7.79	0.86	−0.81
	RF-U	30–60	0.19	9.62	0.79	−0.90
	RF-L	60–100	0.19	10.13	0.78	−0.89
	RF-L	100–200	0.20	10.69	0.76	0.20
Clay	RF-U	0–5	7.23	11.74	0.68	2.75
	RF-U	5–15	6.11	9.95	0.76	2.97
	RF-L	15–30	7.29	10.21	0.75	0.27
	RF-L	30–60	9.29	12.05	0.72	−7.17
	RF-L	60–100	11.36	13.52	0.60	−5.93
	RF-U	100–200	12.99	15.70	0.50	3.90
Sand	RF-U	0–5	10.83	11.16	0.75	0.25
	RF-U	5–15	9.25	10.06	0.83	0.84
	RF-U	15–30	9.16	9.78	0.83	0.04
	RF-U	30–60	9.73	10.09	0.84	2.47
	RF-U	60–100	13.78	13.89	0.67	−0.03
	RF-U	100–200	13.91	14.63	0.71	−3.82

RF-U: random forest model using the Upper set of covariates. RF-L: random forest model trained using the Lower set of covariates.

Trained models outperformed the results of several studies reviewed, where the same properties were mapped at the national and regional scale, like Viscarra-Rossel, *et al*.^63^ (R^2^ between 0.36–0.49 for sand, 0.38–0.46 for silt and 0.39–0.53 for clay), Loiseau, *et al*.^64^ (R^2^ between 0.15–0.45 for clay), Adhikari, *et al*.^65^ (R^2^ between 0.50–0.55 for clay), Ramcharan *et al*.^66^ (R^2^ of 0.57 for sand, 0.46 for clay and 0.42 for bulk density), and only comparable with the results obtained by Akpa, *et al*.^51^ (R^2^ between 0.43–0.91 for clay, 0.51–0.92 for sand, 0.59–0.88 for silt). Furthermore, the performance of the models decreased with depth, which responds to the decreasing sample of points at lower depths.

### Maps of soil physical properties

**Fig. 6:**
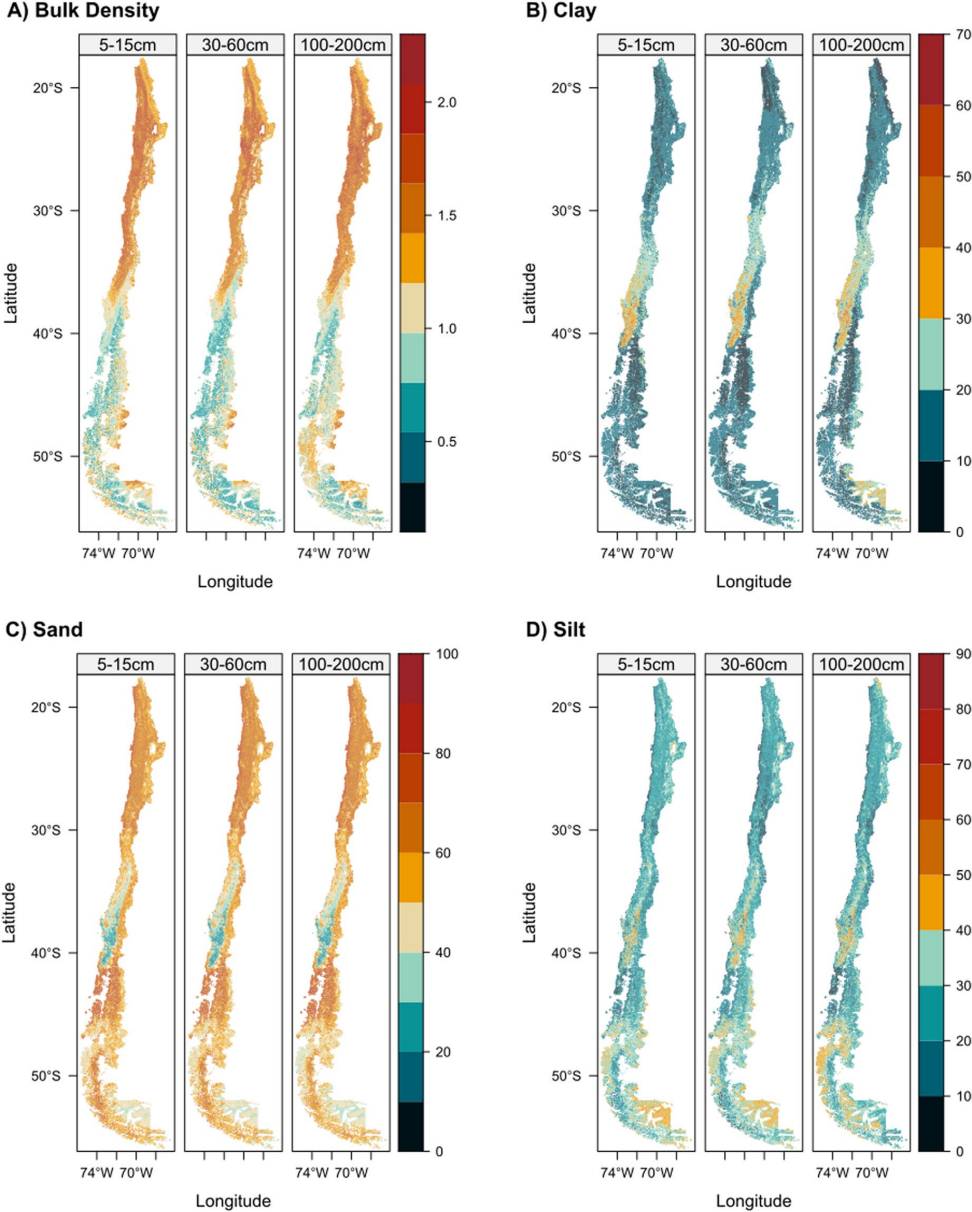
Soil maps for clay, sand and bulk density for depths of 5–15, 30–60 and 100–200 cm.

Figure 6 depicts the spatial distribution of the modeled soil properties for the first horizon, the remaining maps are available on the supplementary information (Figs. S1–4). Sand and clay showed an inverse spatial distribution, as north of 30°S and south of 40°S are the lowest values for clay (~20%) and the highest values for sand (~50%). Between 30 and 40°S, both particle sizes showed more complex patterns with a noticeable north-south gradient with increasing clay and decreasing sand content. By contrast, silt showed a different pattern than the other particles, with a higher content in the central valley, especially around 40°S, and in southern Patagonia. Changes in particle size distribution with depth were subtler than its horizontal variation. The mean clay content decreased from 0 to 30 cm, reaching its maximum values at 100–200 cm (Table 5). Mean sand content was at its minimum value of 15–30 cm and then increased its content towards 60–100 cm. Bulk density showed a noticeable north-south gradient where the maximum values were at the northern part of the country and decreased at lower latitudes. Between 20–30°S, bulk density increased from the Coastal Range from east to west, but the difference was smoother at lower depths. Then, from 30–40°S, a transition zone begins in which values lower than 1 g/cm3 were observed towards the east of the Central Valley and Andes Mountains. At lower latitudes than 40°S, values lower than 1 g/cm3 were generally observed except in some parts of Patagonia. In general, bulk density values increased with depth for the study area except for zones on the insular part of the country at 50°S.

**Table 5:** PICP values for a 90% confidence prediction interval.

Depth	Bulk density	Sand	Clay
0–5	0.98	0.97	0.96
5–15	0.97	0.97	0.98
15–30	0.98	0.96	0.98
30–60	0.97	0.93	0.95
60–100	0.94	0.96	0.95
100–200	0.95	0.95	0.93

**Fig. 7:**
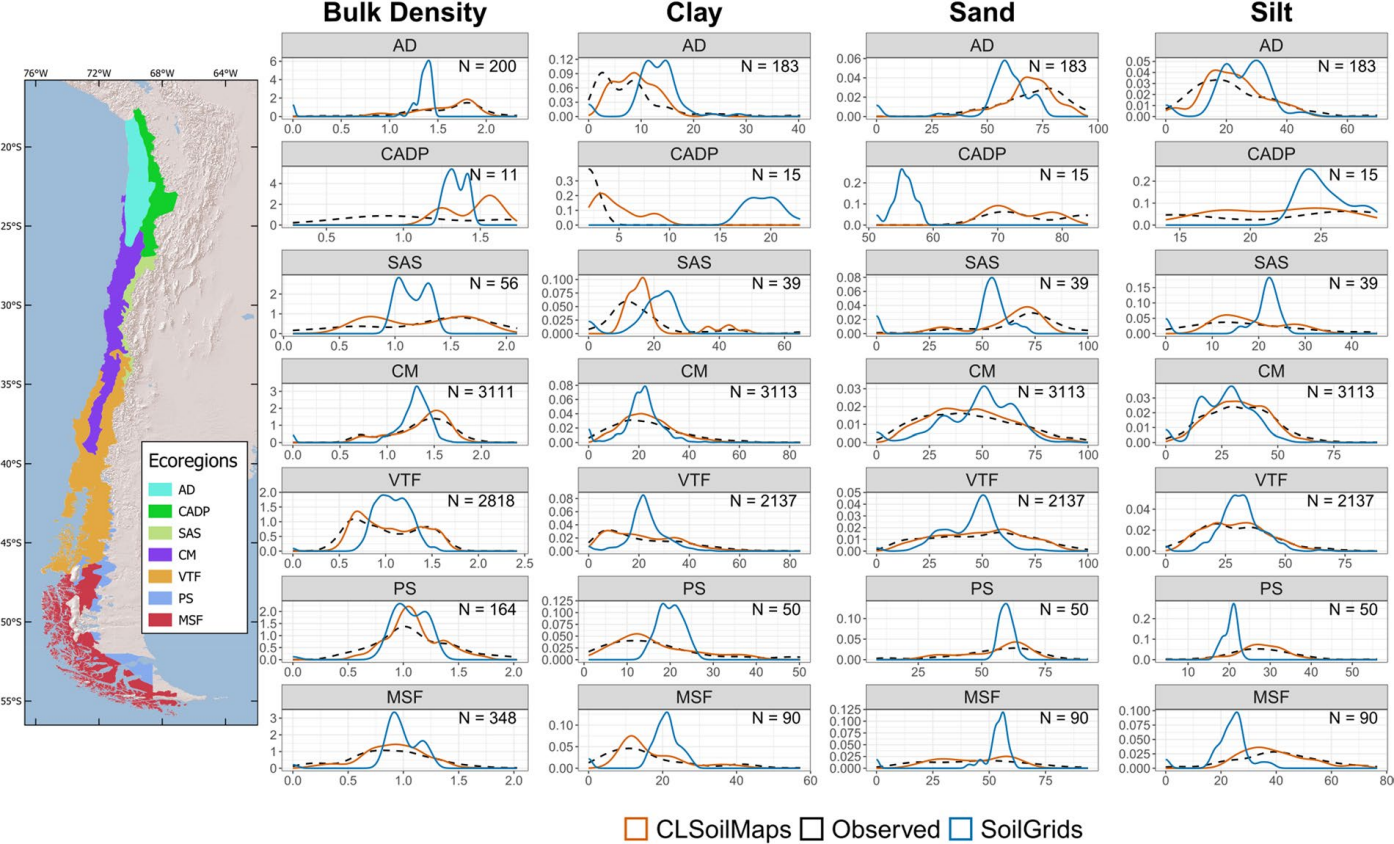
Probability density functions at each ecoregion for clay, silt, sand, and bulk density. AD: Atacama Desert, CADP: Central Andean Dry Puna, SAS: Southern Andean Steppe, CM: Chilean Matorral, VTF: Valdivian Temperate Forests, MSF: Magellanic Subpolar Forests, PS: Patagonian Steppe. The black line is the distribution of observed data, the red line is the predicted value distribution of CLSoilMaps products, and the blue line is the SoilGrids 2.0 predicted values. A map of Ecoregions adapted from Olson *et al*., 2001 is displayed on the left.

Figure 7 shows that the amount of data sampled at each ecoregion was widely heterogeneous with a range from 15 to 3113 samples for textures and 11 to 3111 samples for bulk density. Among these ecoregions, the ones located in the Andes Range and the southern limit of the country were the most underrepresented.

For clay, there is a general overestimation of its content by SoilGrids (SG) throughout all ecoregions, except for the Chilean Matorral, where the peak of the distribution coincides but is higher for SG than for CLSoilMaps and the observed values. By contrast, CLSoilMaps show a similar distribution of clay content to the observed values except at the Central Andean Dry Puna, where contents below 5% are underrepresented. Sand content predicted by our product also shows a similar distribution with the observed values for all ecoregions, even in sparsely sampled areas. SG underestimates sand content in the Atacama Desert and the Andean ecoregions, whereas for the rest, it showed a narrower distribution of predicted values than the observed. Although not modeled directly with a DSM approach, Silt shows a similar distribution to the observed values. The most remarkable differences are in the Central Andean Dry Puna, where CLSoilMaps overrepresents values between 17 and 25% of silt content, and the Atacama Desert, where the model underrepresented silt content below 10%. SG shows an overestimation of silt content in the northern ecoregions, such as the Atacama Desert and the Central Andean Dry Puna, and an underestimation in the southern ecoregions, such as the Patagonian Steppe and the Magellanic Subpolar Forests.

The developed models represent Bulk density values well, capturing observed bimodal distributions like those in the Valdivian Temperate Forest. The most prominent disagreement with the observed values is in the Central Andean Dry Puna, where all predicted values are above 1 g/cm3 for both products, while the observed data showed that most soils are below that threshold. For this soil attribute, SG shows a clear upper boundary at 1.5 g/cm3, which may be a severe limitation for using this product in this area of the country.

### Maps of soil textural classes

**Fig. 8:**
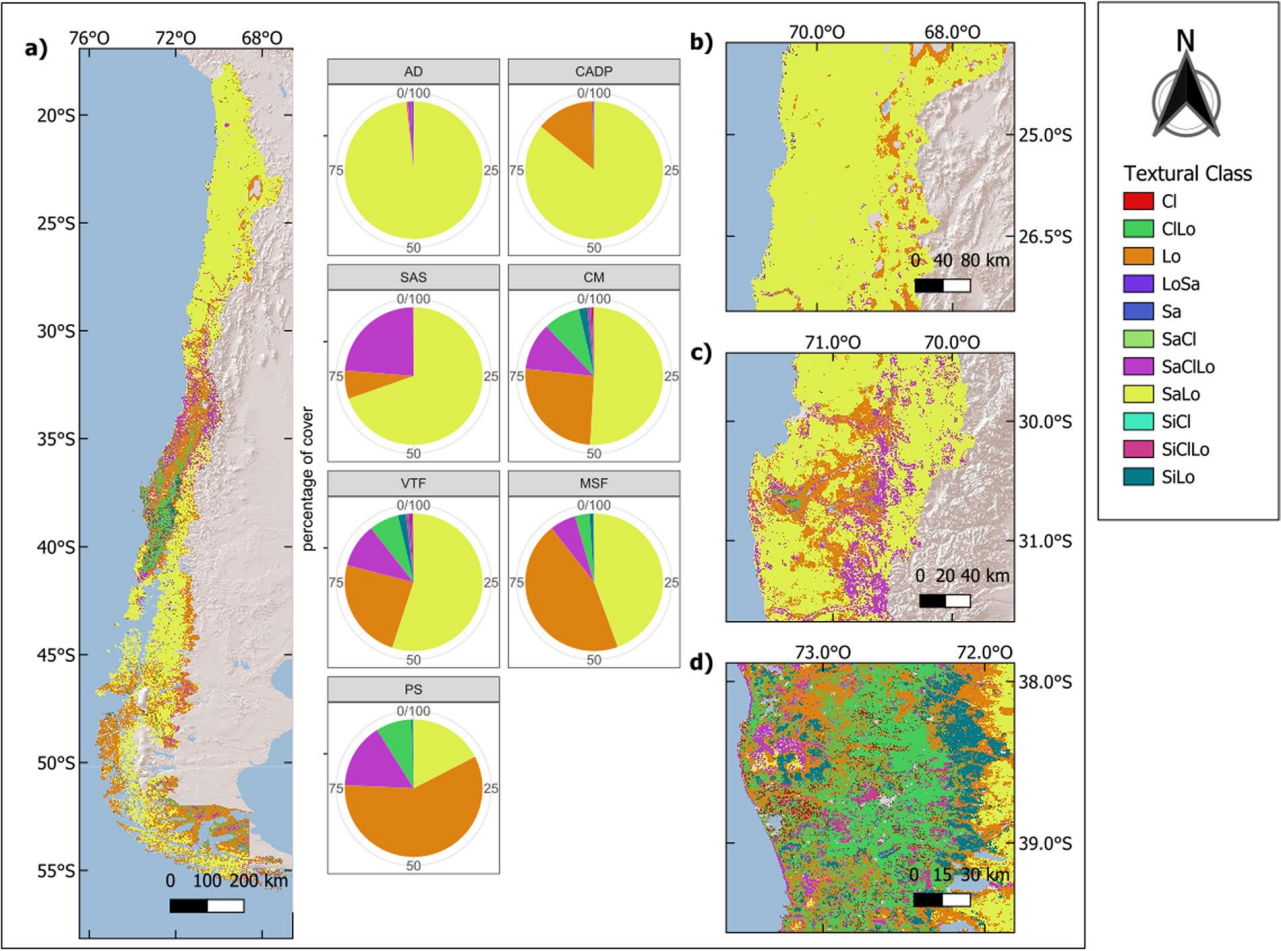
Classification of textural classes for horizon 0–5 cm according to the USDA classification system. CL: clayey; ClLo: Clayey loam, Lo: Loam; LoSa: Loamy sand; Sa: Sandy; SaCl: Sandy clay; SaClLo: Sandy clay loam; SaLo: Sandy loam; SiCl: Silty clay; SiClLo: Silty clay loam; SiLo: Silty loam. Map of textural classes for Chile, pie charts show the percentage of pixels inside the ecoregion that belong to each textural class (**a**). Zoom of the soils on the Atacama Desert where sandy loam soils are dominant (**b**). Zoom of the first valleys with water courses (**c**). Zone with complex soil patterns and appearance of Andic soils (**d**).

Soils with coarser textures, such as sandy loam and loamy sand, are widely represented nationwide (Fig. 8). In the Atacama Desert and the Central Andean Dry Puna, sandy loam soils are dominant, but loamy soils are more frequent, especially in areas around salt flats and wetlands. Following the Andes range to the south in the Southern Andean Steppe, coarse texture soils prevail until 29°S, from which a transition begins to medium texture soils classified as sandy clay loam.

The Chilean Matorral ecoregion shows a transition of soils from coarser textures at its northern limit to finer textures at its southern limit, which results in more complex patterns of soil distribution. In the northern part of the ecoregion, sandy loam and loamy textures still dominate, and from 32°S southwards, there is a mixture of mainly sandy clay loam and loamy soils. Despite this coarser texture dominance, eruptions of fine texture soils appear generally in flat parts of the valleys, such as in the Limarí, Aconcagua or Maipo basin with clay loam and silty loam-textured soils, which become more prominent from 34°S and dominate the landscape from 36°S.

The Valdivian Temperate Forests ecoregion shows a similar distribution of texture classes percentage cover to the Chilean Matorral. This region comprises the coastal range and coastal plains from 34°S and the Andes range from 33°S. The former shows a combination of sandy clay loam and loamy soils, which gradually change to finer texture soils such as clay loam, while the latter presents the inverse pattern with medium texture soils at its northern limit and coarser textures towards its southern limit. Meanwhile, in the central valley, fine and medium texture soils like clay loam, silty clay loam, and silty loam soils are predominant until 41°S, where an abrupt change is observed to coarser texture soils such as loam and sandy loam. These types of soils are widely represented in the southern ecoregions of the Magellanic Subpolar Forests and Patagonian Steppe, where only small aggregations of clayey loam and sandy clay loam soils can be found.

### Maps of soil hydraulic properties

This section presents the map of the total available water capacity for the whole country and different basins located in contrasting ecoregions of Chile. For brevity, maps of field capacity (FC) and permanent wilting point (PWP) obtained from the Van Genuchten soil hydraulic function parameters predicted by Rosetta V3 are displayed in the supplementary information. Also, these maps are available in the CLSoilMaps products.

**Fig. 9:**
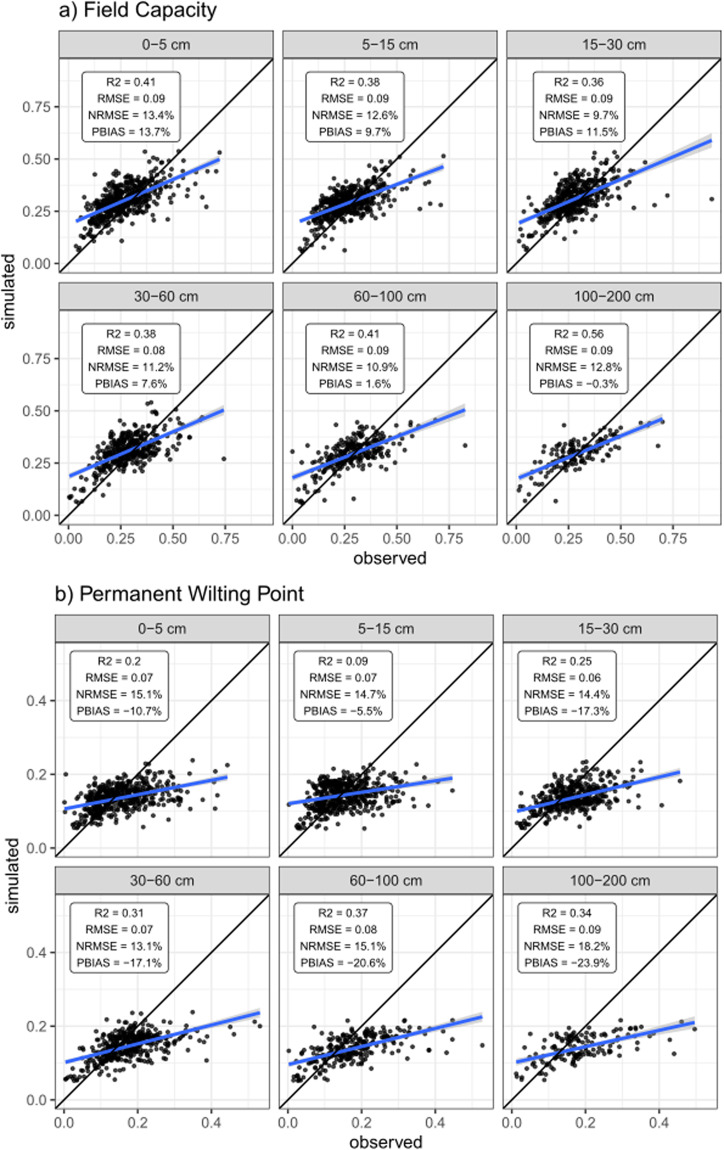
Correlations between observed standardized soil values against field capacity and permanent wilting point.

Validation metrics for FC and PWP are shown in Fig. 9. R2 values for FC are between 0.43–0.65, while PWP R2 values are between 0.17–0.36. Low RMSE values are reported for both properties, with values between 0.09–0.1 for FC and 0.07–0.1 for PWP. Bias metrics show that predictions for PWP tend to underestimate observed values, with PBIAS values between –23.9 to –5.5%. On the other hand, the bias for FC is lower than PWP for all horizons, with values from –0.3 to 13.7%. Concerning depth, unlike physical properties, soil hydraulic properties report better performance with depth, showing the best metrics for the deepest layers.

**Fig. 10:**
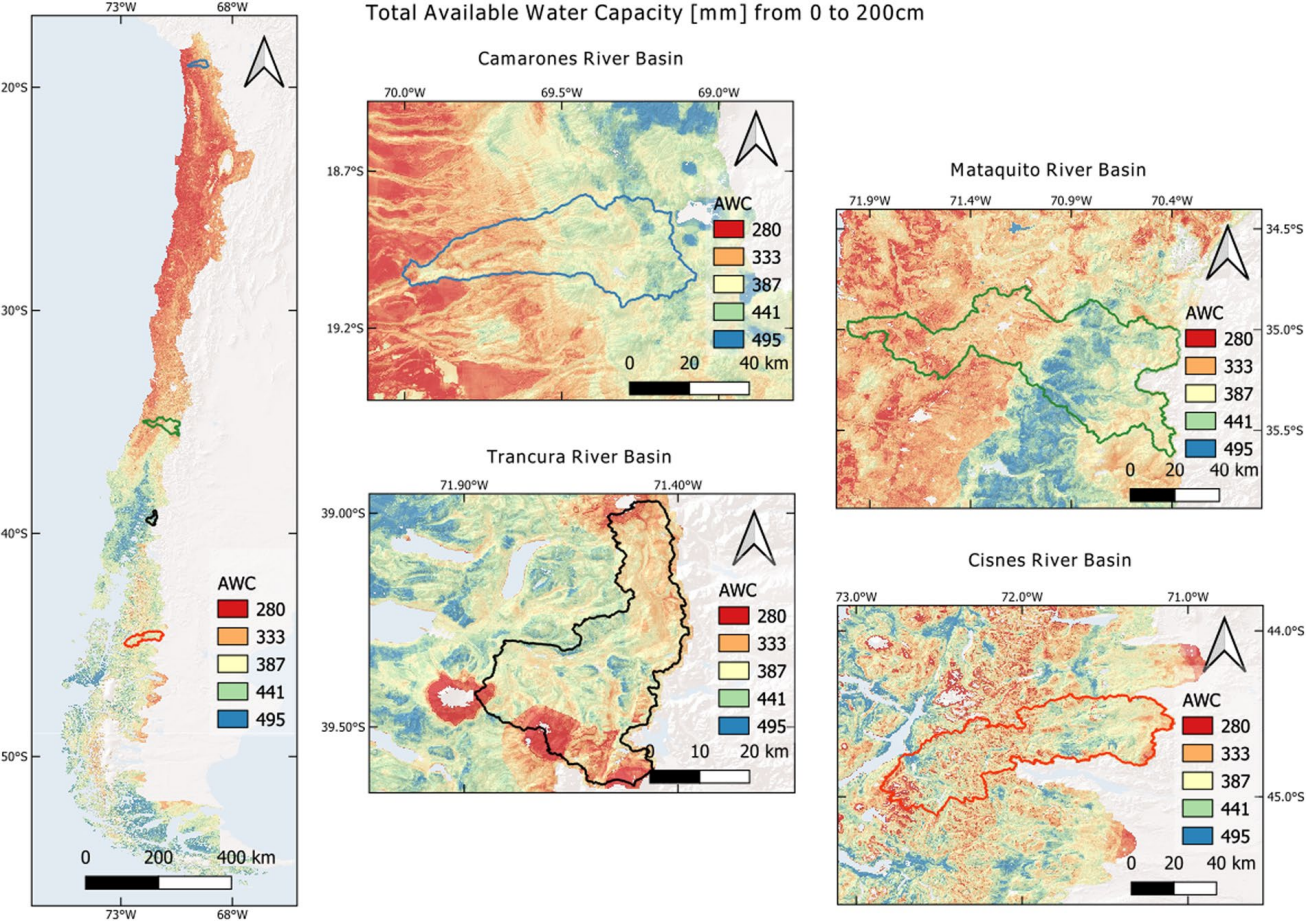
Total available water capacity in mm from 0 to 200 cm. The figure presents a zoom-in for different basins in each macroclimatic zone of Chile.

Available water capacity followed a similar pattern to the physical properties mapped, which are heavily influenced by climate (Fig. 10). AWC was more significant for the central-southern part of the country than the northern part—a crescent gradient in AWC from the northern limit until around 40°S, where the maximum values were observed. From then on, the patterns became more irregular and were determined mainly by topography. At the basin level, the ones located in the northern part of the country revealed greater AWC values at higher altitudes and lower values at the lower parts of the slope. On the other hand, the Trancura basin showed the highest AWC values at lower altitudes with minimums around the volcanoes. At the Cisnes basin, the greatest AWC values were east of the Andes Mountains, where flat lands begin.

### Maps of prediction uncertainty

**Fig. 11:**
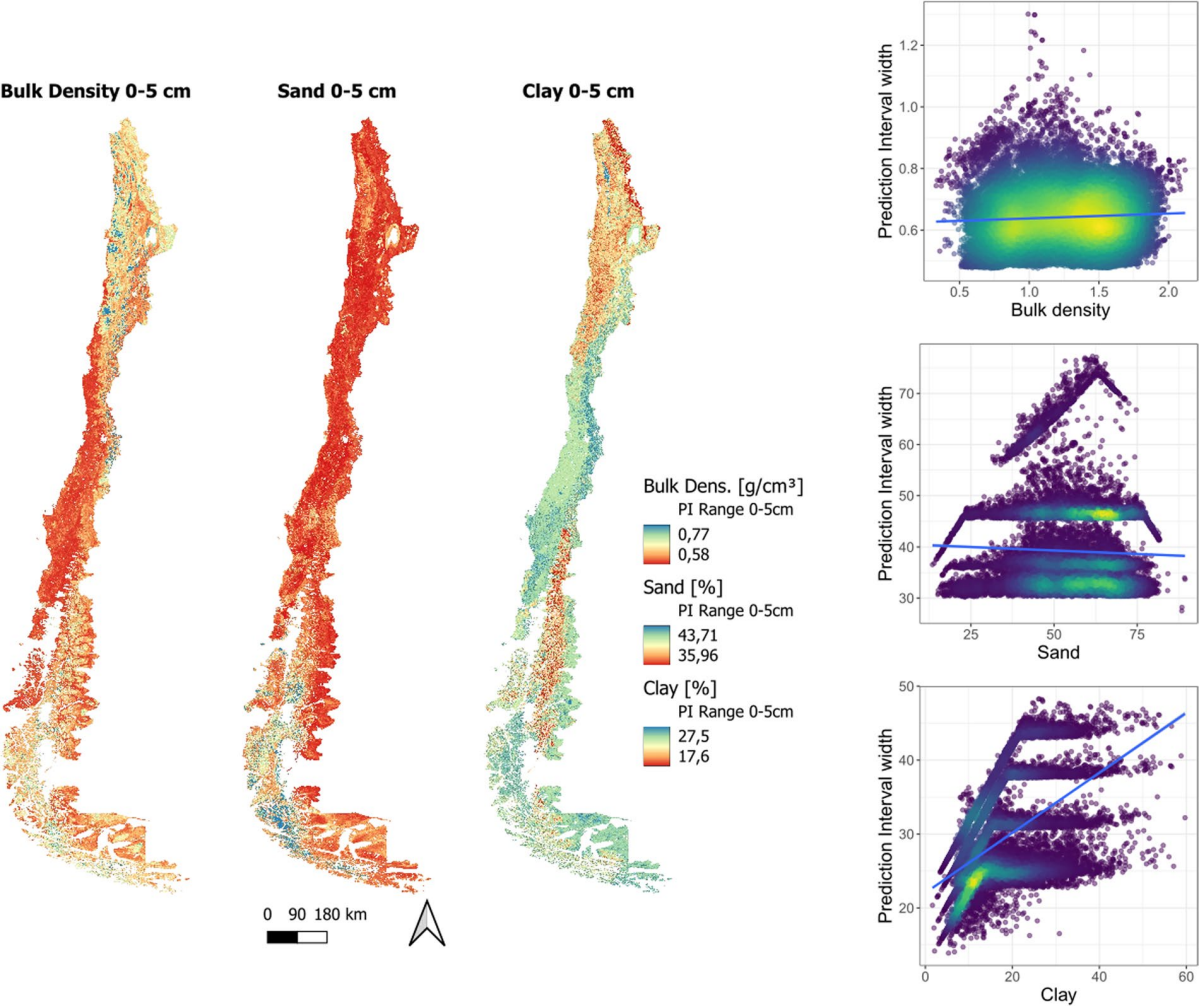
Maps of prediction uncertainty for the first standardized horizon (0–5 cm) for bulk density, clay and sand content (**a**). Plots of prediction interval width and soil attributes (**b**). The blue line is the linear regression between the modeled soil property and its corresponding prediction interval width.

Maps of predicted uncertainty are displayed using the width of the 90% confidence prediction interval (Fig. 11). Wider intervals represent more uncertainty in the prediction mean. The mean prediction interval (PI) width for clay goes from 23.46–36.95%, for sand 32.02–51.91%, and for bulk density 0.54–0.75 (Details in Table 6). Maximum PI width is at the lowest depth for the three soil properties modeled, showing increasing uncertainty with depth. Nevertheless, the models show low IQR for clay 2.59–11.2, sand 1.13–12.09, and bulk density 0.05–0.09 PI width, indicating that the model predictions were stable across different regions. Bulk density showed the lowest PI width in the central part of the country, where soils were more densely sampled. Also, uncertainty increased where the values were closer to the physical limits for bulk density, as seen in the country’s north and south. By contrast, sand showed a stable uncertainty distribution across the entire country, except for the Patagonian archipelago, where PI width becomes larger. Finally, clay predictions showed higher uncertainty in the central and southern regions of the country, which was correlated with higher clay contents Table 7.

**Table 6:** Soil map descriptive statistics for each soil attribute modeled.

Soil Attribute	Depth (cm)	Mean	Median	SD	Max	Min	IQR	1st Quantile	3rd Quantile
Clay	0–5	15.04	12.89	7.27	60.60	1.43	10.42	9.34	19.75
	5–15	15.72	13.52	7.46	74.37	0.69	8.33	10.47	18.80
	15–30	14.93	13.65	6.45	59.73	0.99	7.93	10.28	18.21
	30–60	15.87	14.20	7.22	62.72	0.90	8.49	10.90	19.39
	60–100	16.54	14.72	7.74	69.58	0.48	8.81	11.35	20.16
	100–200	16.83	14.02	8.48	73.86	0.67	11.89	10.47	22.37
Sand	0–5	55.70	56.68	11.93	94.00	4.83	18.08	47.33	65.41
	5–15	56.28	57.36	11.59	95.01	6.20	16.36	48.22	64.58
	15–30	55.72	56.68	11.88	93.75	4.99	18.04	47.39	65.43
	30–60	56.01	57.03	12.10	94.61	4.69	18.05	47.53	65.57
	60–100	56.34	57.51	12.20	94.95	4.68	17.57	47.92	65.49
	100–200	55.75	56.84	12.42	94.92	5.62	18.97	46.51	65.48
Silt	0–5	29.28	28.92	9.22	76.60	0.00	12.83	22.80	35.63
	5–15	28.08	27.91	9.34	73.84	0.00	13.00	21.68	34.68
	15–30	29.32	29.25	9.30	76.60	0.00	14.22	22.29	36.51
	30–60	28.15	28.20	9.40	74.13	0.00	13.97	21.26	35.23
	60–100	27.18	27.13	9.40	75.07	0.00	13.74	20.42	34.17
	100–200	27.53	26.93	9.93	83.76	0.00	13.38	20.79	34.17
Bulk Density	0–5	1.14	1.13	0.37	2.20	0.14	0.66	0.79	1.45
	5–15	1.22	1.21	0.34	2.10	0.23	0.58	0.95	1.52
	15–30	1.13	1.12	0.38	2.20	0.14	0.67	0.78	1.44
	30–60	1.16	1.15	0.36	2.18	0.14	0.66	0.81	1.47
	60–100	1.21	1.20	0.35	2.15	0.24	0.61	0.93	1.54
	100–200	1.24	1.23	0.35	2.14	0.31	0.59	0.97	1.57

**Table 7:** Statistics of map prediction uncertainty.

Soil Property	Depth	Mean	Median	SD	Max	Min	IQR	1st. Quant.	3th. Quant.
Clay	0–5 cm	23.46	24.36	2.6	64.08	13.41	3.89	21.45	25.34
	5–15 cm	24.74	24.21	3.09	62.34	11.67	2.62	23.4	26.02
	15–30 cm	23.76	24.54	2.3	57.72	13.01	2.59	22.5	25.09
	30–60 cm	28.77	29.99	3.31	60.58	16.2	4.94	26.38	31.32
	60–100 cm	33.58	33.97	4.33	62.76	19.19	7.58	30.28	37.86
	100–200 cm	36.95	35.99	5.73	65.98	22.09	11.2	32.12	43.32
Sand	0–5 cm	37.48	36.87	1.88	88.98	23.08	1.35	36.4	37.75
	5–15 cm	33.72	32.93	2.52	92.4	22.23	2.45	32.03	34.48
	15–30 cm	32.02	31.56	1.61	67.9	20.65	1.43	31.02	32.45
	30–60 cm	34.02	33.42	1.9	89.85	21.16	1.5	32.9	34.4
	60–100 cm	46.79	46.4	1.63	98.79	27.66	1.13	46	47.14
	100–200 cm	51.91	47.05	8.56	99.04	28.97	12.09	46.5	58.59
Bulk Density	0–5 cm	0.64	0.63	0.05	1.56	0.47	0.05	0.6	0.66
	5–15 cm	0.55	0.54	0.05	1.62	0.48	0.06	0.51	0.57
	15–30 cm	0.54	0.53	0.05	1.5	0.43	0.06	0.5	0.57
	30–60 cm	0.66	0.65	0.04	1.62	0.49	0.05	0.63	0.68
	60–100 cm	0.72	0.69	0.07	1.87	0.64	0.09	0.66	0.76
	100–200 cm	0.75	0.73	0.07	1.83	0.67	0.08	0.7	0.79

## Usage Notes

Validation metrics showed moderate to good results for all soil attributes and depths, but some limitations must be considered when using these maps for different contexts. Careful consideration must be taken with the scale of the study area since precision at the local scale decreases as the extent of the soil maps is larger^67^. For this reason, it is recommended that when selecting a soil map product, an independent evaluation must be done, either by collecting new soil samples or comparing them with available conventional soil maps.

Another consideration is that the uncertainty of soil maps is different for different regions and even for different soil attributes. As seen on the uncertainty maps (Figure 11), bulk density, sand, and clay show different patterns in their uncertainty prediction that respond to the soil sample density and the range of possible values that the soil property can present in different zones. Even so, the PICP values above 0.9 showed that this uncertainty is overestimated, which means that prediction intervals are too broad. Future work must be done to improve the uncertainty evaluation of the predictions, either by collecting more samples in low-density sampled areas, which has been found to have a great impact on the performance of DSM models^68^, or by testing more direct methods for estimating prediction uncertainty like quantile regression forest^69^.

Furthermore, as demonstrated by the validation metrics, the performance of the models decreases with depth, so using these soil maps for applications where deep soil layers are relevant must be done with care. Moreover, these maps consider the same configuration of SoilGrids products with a fixed maximum depth of 200 cm for all soils, which is only sometimes valid. For instance, soils in the northern part of the country tend to be shallower, while soils to the south have been found to exceed that two-meter limit. This issue is especially relevant when working in forestry or with hydrological applications, where total available water capacity is directly dependent on depth, so considering deeper soils (e.g., in the north) may lead to an important overestimation or, on the contrary, to an underestimation in areas where soils are more profound than 200 cm. To tackle this disadvantage, an evaluation of global soil maximum depth products like the ones provided by SoilGrids could be done. Future efforts must be made to sample soils at their maximum depth so that this constraint can be incorporated into soil maps for Chile.

### Supplementary information

Suplementary Information

## Data Availability

Source code of this project can be accessed at CLSoilMaps Github repository located at https://github.com/diegodinamarca/CLSoilMaps.git.
