# Supplementary material for: CLSoilMaps: A national soil gridded database of physical and hydraulic soil properties for Chile
Source: Sci Data. 2023 Sep 16;10:630. doi: 10.1038/s41597-023-02536-x (PMC10505153; doi:10.1038/s41597-023-02536-x)

# Supplement

Table S1. Database summary statistics of soil properties modeled

| **Soil Attribute** | **Clay (%)** | **Sand (%)** | **Bulk Density (g/cm^3^)** |
| --- | --- | --- | --- |
| Maximum | 85.00 | 100.00 | 84.60 |
| Minimum | 0.00 | 0.26 | 0.00 |
| Mean | 22.30 | 47.39 | 30.30 |
| Median | 19.00 | 47.26 | 29.50 |
| St. Dev. | 15.70 | 23.27 | 15.25 |
| Soil Profiles | 4466 | 4466 | 4466 |
| N | 1212 | 1212 | 1212 |

**Figure S1.** Clay predictive maps for six standard horizons.

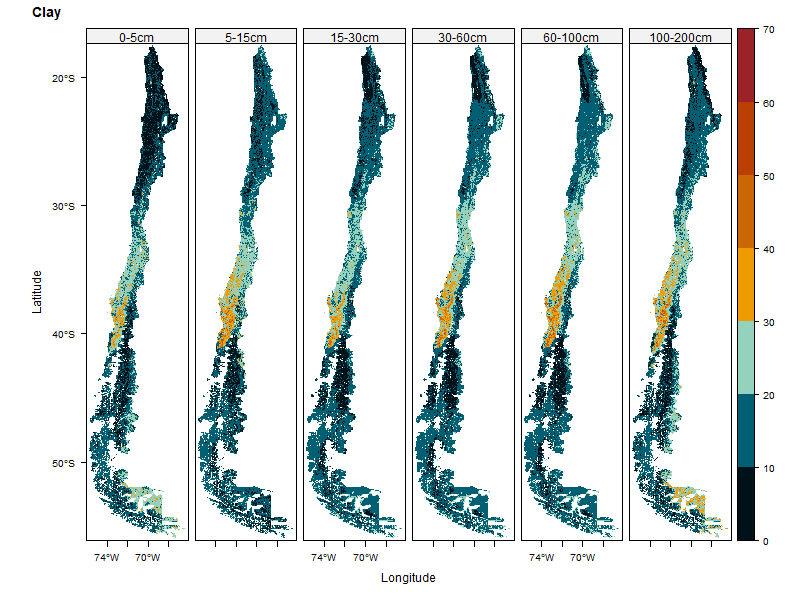


**Figure S2.** Sand predictive maps for six standard horizons.
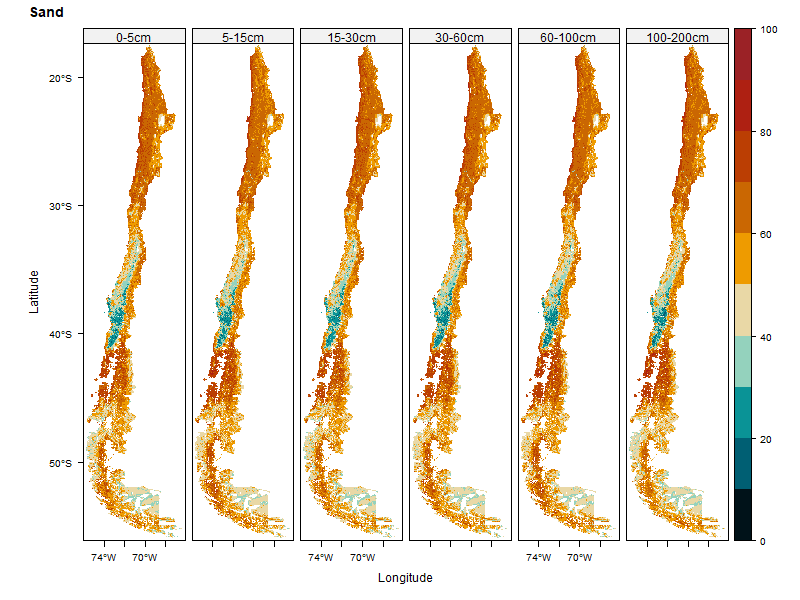


**Figure S3.** Bulk Density predictive maps for six standard horizons.
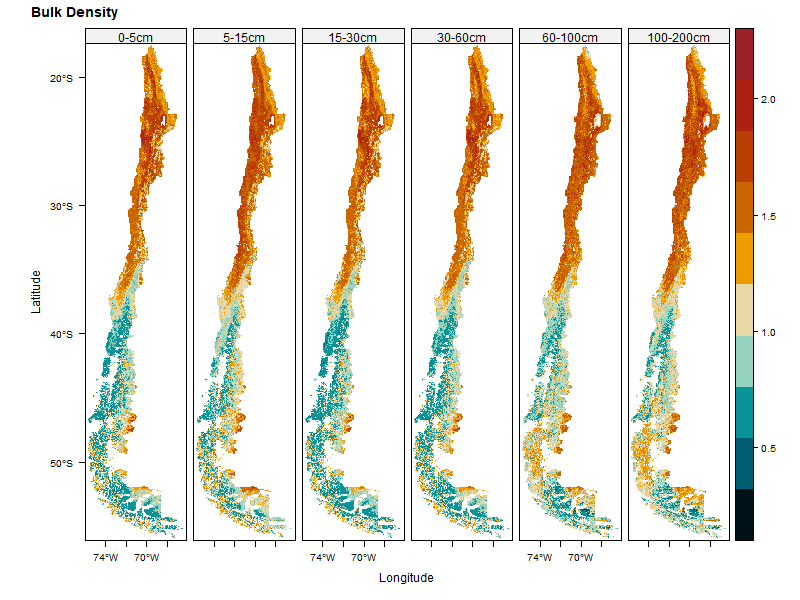


**Figure S4**. Silt predictive maps for six standard horizons.


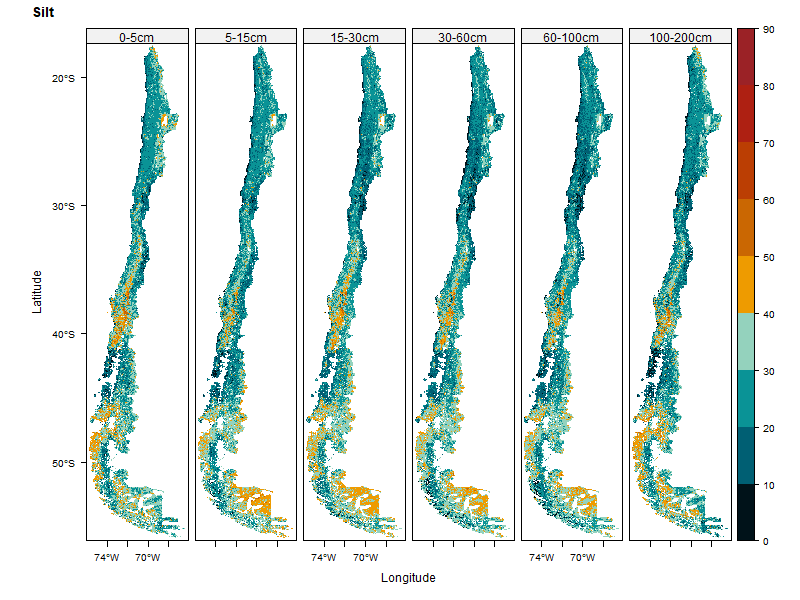


**Figure S5**. Field Capacity predictive maps for six standard horizons.


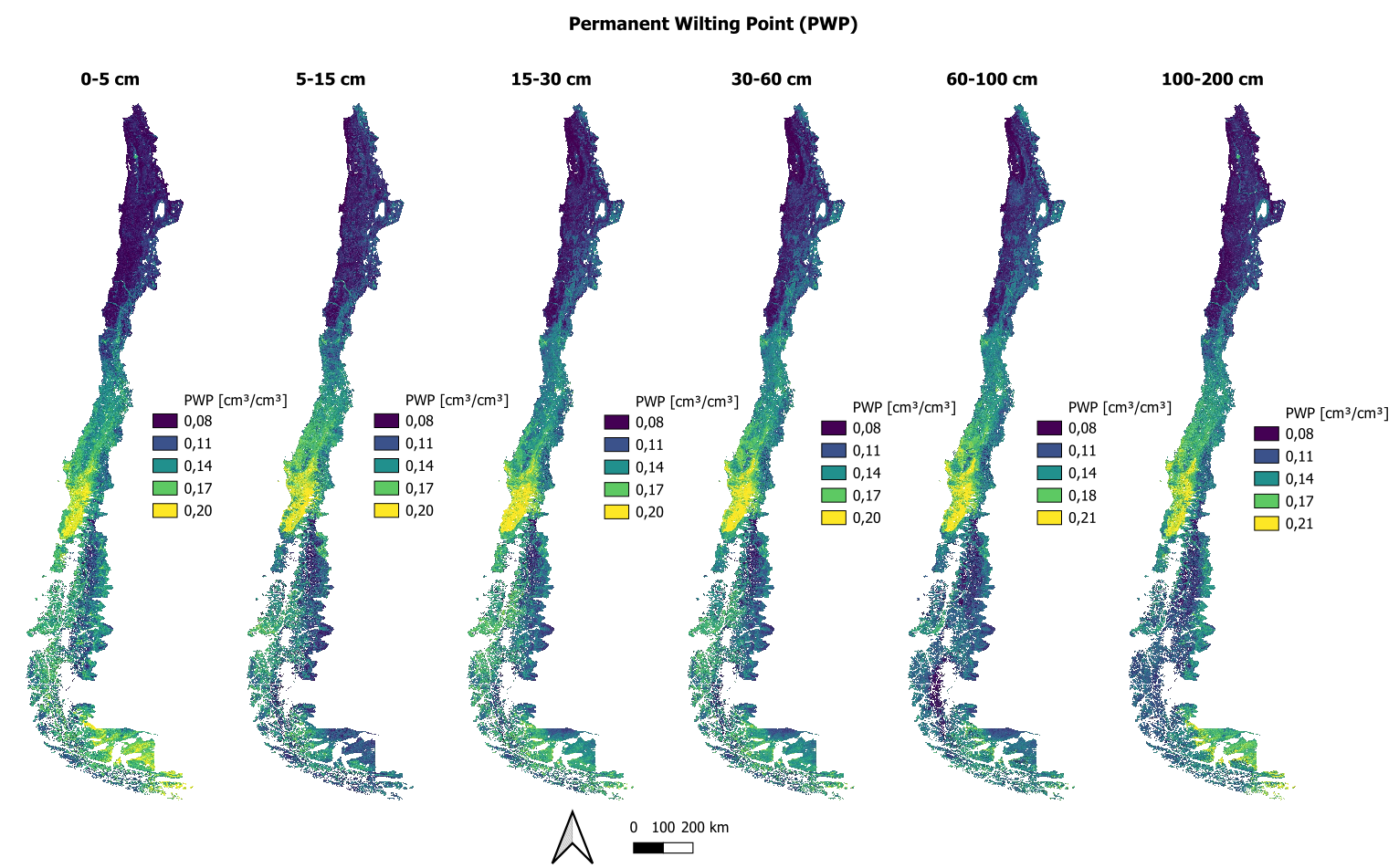


**Figure S6**. Permanent Wilting Point predictive maps for six standard horizons.


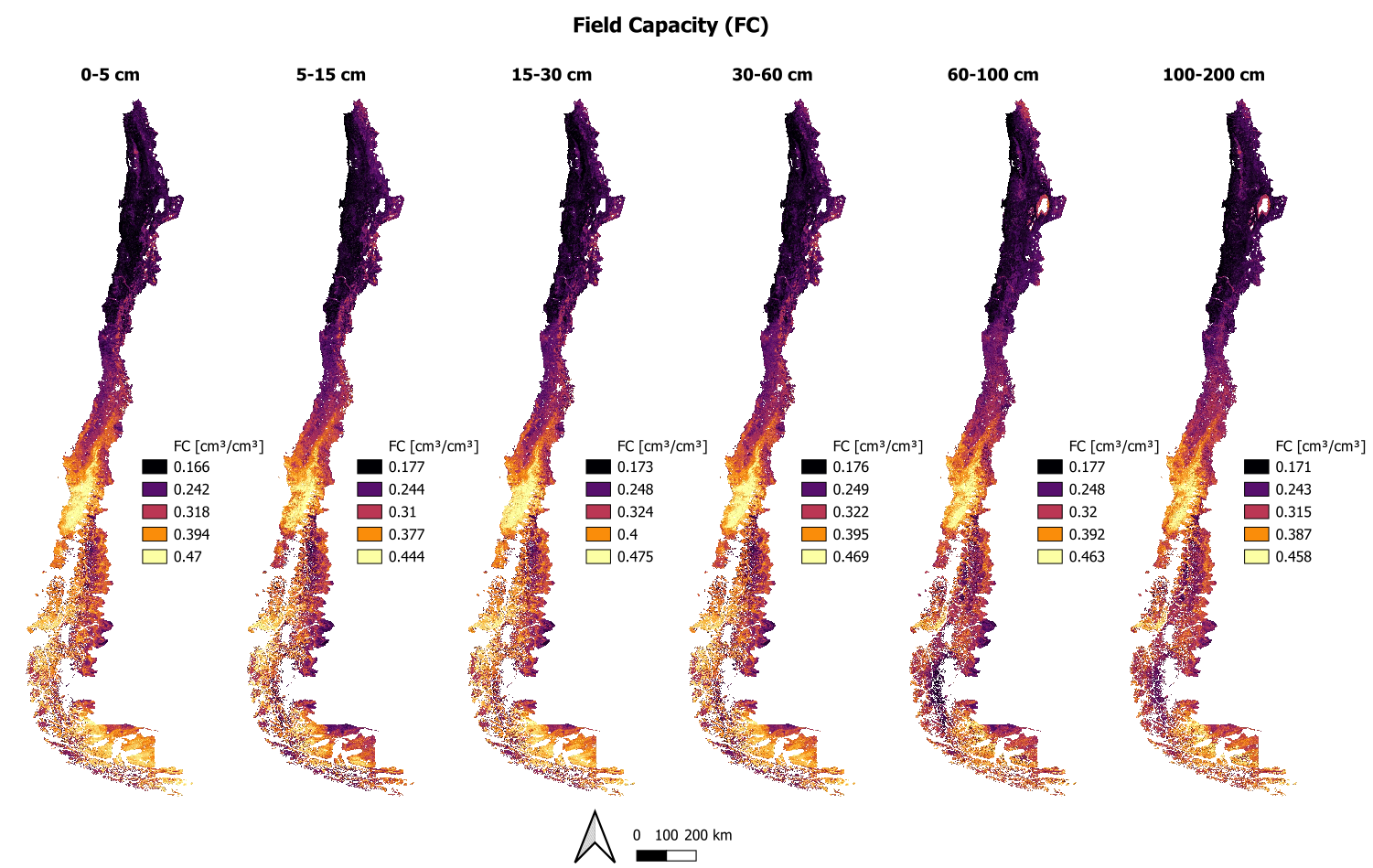

Supplement: Supplementary file 1 — Suplementary Information [file 41597_2023_2536_MOESM1_ESM.docx]
